# 4219 Discrepancies in flavor preferences among adult ever users of various tobacco products in the US – Findings from The Population Assessment of Tobacco and Health Study (2015-2016)

**DOI:** 10.1017/cts.2020.175

**Published:** 2020-07-29

**Authors:** Liane M Schneller, Dongmei Li, Zahíra Quiñones-Tavárez, Maciej Goniewicz, Amanda Quisenberry, Zidian Xie, Irfan Rahman, Scott McIntosh, Richard O’Connor, Deborah J. Ossip

**Affiliations:** 1 University of Rochester Medical Center

## Abstract

OBJECTIVES/GOALS: Flavorings differ between brands and tobacco products, potentially altering the sensory perceptions. This study aimed to examine discrepancies in flavor preference across various non-cigarette tobacco products among a national representative sample of US adult regular tobacco users. METHODS/STUDY POPULATION: Data from the Population Assessment of Tobacco and Health (PATH) Study Wave 3 (W3) were used. Weighted prevalence of flavor preference for various tobacco products, including electronic nicotine delivery systems (ENDS), traditional cigars, cigarillos/filtered cigars, hookah and snus/smokeless, was presented for 9,037 adult current and new former users of multiple flavored tobacco products. Within-subject flavor discrepancies were assessed using generalized estimating equations (GEE) models considering the complex sampling design of the PATH study. RESULTS/ANTICIPATED RESULTS: Most regular users of a flavored tobacco products reported using one flavor category per product. Fruit flavors, followed by tobacco, were the most common flavor categories among ENDS (32% and 25%, respectively) and hookah users (44% and 36%, respectively). Tobacco flavor was the most common among regular users of traditional cigars (80%), cigarillos/filtered cigars (55%), and smokeless tobacco (79%). Polytobacco users of ENDS and traditional cigars had the largest discrepancy, where about 68-76%% used different flavor categories when switching products. Conversely, polytobacco users of traditional cigars and cigarillos/filtered cigars had the lowest discrepancy (23-25%). DISCUSSION/SIGNIFICANCE OF IMPACT: Many consumers of multiple tobacco products had different flavor preferences when switching between products. In the event of a partial or full flavor ban for ENDS, these findings raise questions about consumer loyalty to a particular tobacco product or a particular flavor category. Conflict of Interest Description: MLG serves as a paid consultant for Johnson & Johnson and has received research grant from Pfizer, manufacturers of smoking cessation medications. The other authors have no conflicts to declare. CONFLICT OF INTEREST DESCRIPTION: MLG serves as a paid consultant for Johnson & Johnson and has received research grant from Pfizer, manufacturers of smoking cessation medications. The other authors have no conflicts to declare.

